# Effect of Nano-TiO_2_ Addition on Some Properties of Pre-Alloyed CoCrMo Fabricated via Powder Technology

**DOI:** 10.3390/ma19010186

**Published:** 2026-01-04

**Authors:** Jawdat Ali Yagoob, Mahmood Shihab Wahhab, Sherwan Mohammed Najm, Mihaela Oleksik, Tomasz Trzepieciński, Salwa O. Mohammed

**Affiliations:** 1 Department of Oil Equipment Inspection and Welding Techniques, Kirkuk Polytechnic College, Northern Technical University, Kirkuk 36001, Iraq; jaw209662@ntu.edu.iq; 2 Consultant Orthopaedic Revision and Complex Knee Surgeon, Medical City Complex, Ministry of Health, Baghdad 10011, Iraq; afwahab2003@yahoo.com; 3 Department of Fuel and Energy Engineering Techniques, College of Oil & Gas Techniques Engineering-Kirkuk, Northern Technical University, Kirkuk 36001, Iraq; 4 Faculty of Engineering, “Lucian Blaga” University of Sibiu, Victoriei Bd. 10, 550024 Sibiu, Romania; mihaela.oleksik@ulbsibiu.ro; 5 Department of Manufacturing Processes and Production Engineering, Faculty of Mechanical Engineering and Aeronautics, Rzeszów University of Technology, al. Powstańców Warszawy 8, 35-029 Rzeszów, Poland; tomtrz@prz.edu.pl; 6 Department of Computer Systems Techniques, Kirkuk Polytechnic College, Northern Technical University, Kirkuk 36001, Iraq; salwa@ntu.edu.iq

**Keywords:** CoCrMo alloys, powder metallurgy, dry sliding wear, wear debris, nano-TiO_2_

## Abstract

The CoCrMo alloys are progressively utilized as biomaterials. This research is dedicated to studying the consequence of (1, 3, and 5) wt% nano-TiO_2_ addition on the porosity, microstructure, microhardness, and wear behavior of pre-alloyed CoCrMo powder produced by powder metallurgy (PM). Microstructural features were examined using SEM, SEM mapping, and XRD. Wear behavior was assessed through pin-on-disk tests performed under dry sliding conditions at varying loads and durations. Porosity increased with the addition of nano-TiO_2_, from 15.26 at 0 wt% reaching 25.12% at 5 wt%, while density decreased from 7.16 to 6.33 g/cm^3^. Microhardness exhibited a slight improvement, attaining 348 HV at 5 wt%. SEM and XRD analyses confirmed partial particle separation after sintering and identified the TiO_2_ reinforcement as rutile. Wear tests revealed that adding 1 wt% nano-TiO_2_ enhanced wear resistance, whereas extended sliding durations resulted in increased wear rates. Adhesive wear was the predominant mechanism, accompanied by limited abrasive wear, oxidation, and plastic deformation.

## 1. Introduction

CoCrMo alloys (CCMAs) are employed in various industrial applications, including bearings, engine components, wind and gas turbines, aeroengines, and nuclear systems, due to their superior bulk mechanical properties, such as strength, wear resistance, and commendable corrosion resistance [1,2,3]. Among all implant materials, CCMAs achieve the optimal equilibrium regarding fatigue, strength, wear resistance, and corrosion resistance [4,5]. CCMAs were employed at multiple anatomical sites to augment and optimize the efficacy of health-related functions in humans [6]. The biomedical applications encompassed the manufacturing of shoulder prostheses, orthopedic prosthetic devices, wear-resistant coatings, synthetic knee and hip joints, and fracture-resistant devices [7,8]. Products composed of CCMAs can be manufactured through hot forming, casting, and the powder technology route (PTR) [1,9,10,11,12]. Oksiuta et al. [13] mentioned that bioglass (BG) with 10 wt% unusually upsurges the mechanical properties along with the resistance to corrosion of the CCMA. Abbass et al. [14] investigated the wear performance and mechanism of CCMA, which is composed of PTR. The main wear mechanism was an adhesive type. The plastic deformation and oxidation natures are also evident from the morphology of the worn surface, as observed under scanning electron microscopy (SEM). The mechanisms of friction are complex, involving several interacting processes at the microscopic scale. However, for dry friction, the two primary physical mechanisms are generally recognized as adhesion and asperity deformation (plowing).

Abbass et al. [15] explored the effect of α-Al_2_O_3_ nanoparticle (NP) additions on some properties of CCMA made up by PTR. The hardness decreased with the increase in NPs, primarily at 5 wt% α-Al_2_O_3_. On the other hand, poverty increased due to the addition of NP. In other work, Abbass et al. [16] investigated the influence of Al_2_O_3_ NPs on the corrosion performance of biomedical CCMA in Ringer’s liquid. They concluded that the addition of α-Al_2_O_3_ NPs enhances the corrosion resistance of the alloy. The addition is successfully achieved by the fabrication of the testing samples via PTR. To further enhance the properties of CCMAs, numerous investigators have dedicated their studies to investigating the effects of various metallic additives. Cui et al. [17] studied the result of adding silver to the wear performance of auto-lubricated Co-alloys at high temperatures made up by powder metallurgy. This is in line with Al-Deen et al. [18], who studied the CoCrMo (F75) properties after doping with yttrium. Haleem et al. [19] tested the properties of CoCrMo alloys (F75) once they were doped with Ge.

Further efforts by attentive investigators aimed to understand the influence of micro-sized ceramic additives on the properties of CCMAs. Abdullah et al. [20] reported a 25% increase in microhardness when 5% BG was added. Grądzka-Dahlke et al. [21] pointed out that the development of tribological properties for composites can be understood by incorporating dissimilar hard, wear-resistant particles, such as TiO_2_, SiO_2_, Si_3_N_4_, and B_4_C, to strengthen the metallic matrix. Tian et al. [22] noted that NPs have been well-thought-out as an eye-catching family of materials over the past few years due to their innovative properties that do not exist in the bulk. The exceptional characteristics of NPs stem from their small sizes and large specific surface areas. Tok et al. [23] specified that Al_2_O_3_ and TiO_2_ are currently among the most valuable oxides, as they have been utilized in various engineering sectors, including abrasive grains, heat-resistant materials, coatings, advanced ceramics, and cutting materials. Moreover, TiO_2_ NPs are cost-efficient and non-hazardous, high-surface/volume biocompatible material permitted for use widely in biomedical applications, and they are a drug and food-connected product by the United States Food and Drug Administration USFDA [24,25].

For a prolonged search on the World Wide Web, the remark was about the absence of investigations into the impacts of TiO_2_ NPs on the CCMA properties. This was one of the stimuli to achieve recent research. Consequently, the goal was to investigate the effect of nano-TiO_2_ on specific CCMA properties, as defined by PTR.

## 2. Materials and Methods

### 2.1. Materials

Pre-alloyed CCMA powder with spherical form was used as a major constituent with chemical composition that was tested by Kosgeb laboratory for metal quality control in Turkey consisted of 27.056 wt% Cr, 6.1695 wt% Mo, 0.5285 Mn, 0.182 wt% Fe, 0.147 wt% Ni, 0.0767 wt% Al, 0.048 wt%, 0.036 wt% C, Si, 0.0275 wt% V, 0.0105 wt% Al and other elements with minor amounts which are W, Ti, Pb, As, Sn, P and S. The reminder was cobalt. The particle size of CCMA powder is arranged between 25 and 45 µm. Nano-TiO_2_ powder, rutile type, with purity of 99.9% as stated by the producer, was used as nano-addition, and its particle size distribution was (83.4) nm, analyzed using the NanoBrook 90Plus Particle Size Analyzer instrument/Brookhaven Instruments Corporation, Holtsville, NY, USA.

### 2.2. Experimental Procedures

CCMA powder was dried at 120 °C for 15 min prior to mixing with 3 wt% of stearic acid powder, which was added to every charge to facilitate the pressing process. Mixing and milling of charge combinations were carried out according to the steps outlined in references [14,26]. Thereafter, a group of powder mixtures was equipped. The group comprises 3 subcategories (T1, T2, and T3) for which 1, 3, and 5 wt% nano-TiO_2_ were added to the CCMA base powder, respectively, and individually. A pressing pressure of 1000 MPa was applied via a hydraulic press at room temperature. The pressing pressure was controlled using the UIY8 intelligent pressure gauge. The green compacts (GCs) were thermally treated in an electric CARBOLITE-type furnace. A sintering cycle was performed in two steps within an argon gas atmosphere. Initially, GCs were heated to the stearic acid elimination temperature (500 °C) for 120 min, followed by heating and soaking for another 120 min at 1200 °C. Sintering was performed at (1000, 1100, 1200, and 1300) 1200 °C in the previous work [26]. According to the higher microhardness value achieved at 1200 °C, this temperature is considered the optimal sintering temperature for the present work. The furnace turned off, and the specimens were left to cool gradually inside it. Green densities (ρg), theoretical density (ρTh) for GCs, and total porosities (TPs) of the studied CCMA nano-TiO_2_ composites are determined using the widely used mathematical expression, as referred to in [15,26] and listed below:

ρg = Mc/Vg(1)

ρTh = Ʃ(ρM × XM)(2)

PT% = (1 − ρg/ρTh) × 100(3)

Vickers microhardness for sintered compacts (SCs) was evaluated using a Metkon-type microhardness device with a 500 g test load, which is compatible with ASTM E-384 [27]. The number of readings that were extracted from each test was five. Optical microscopy using the OPTIKA microscope/OPTIKA Srl, Ponteranica Bergamo, Italy from Italy, SEM-EDS (Energy Dispersive X-ray Spectroscopy), and mapping via the TESCAN device, Tescan Orsay Holding, Brno, Czech Republic were performed. Moreover, X-ray diffraction (XRD) analysis was performed using a Shimadzu XRD-6000 laboratory diffractometer/Shimadzu Corporation, Kyoto, Japan. The formally listed procedures were stated in the description stage, including the analysis of the utilized powders, phase analysis, and their identification.

### 2.3. Dry Sliding Wear Test

The analogous faces of the 10 mm diameter specimens were smoothed using five different SiC emery papers with (240 to 3000) grit under a stream of water, then polished using suspension solution of water/5 µm alumina, cleaned up with distilled water, and dried inside an oven LHT/60-type at 120 °C for 0.5 h. The roughness of the surface of specimens exposed to the wear test was measured using the Pocket Surf^®^ transportable gauge for surface roughness/Nanovea Inc., Irvine, CA, USA. The measurement was performed via a 0.75 mm cut-off length with a 1 mm sampling length. The average of the 3 measurements of the arithmetical mean deviation (Ra) was determined. It is vital to measure the surface morphology using the most commonly used quantification parameter, surface roughness (Ra). This parameter is significant to offer guidance signs for filopodia-driven adhesion [28,29].

All specimens were inspected and then dehydrated in an electric oven at 120 °C for 15 min, followed by gradual cooling to room temperature. They were subsequently sheltered with layers of dried textile and then stored inside plastic ampules, free from moisture. Finally, the specimens were discarded just before the wear test. It is essential to note that the removal of moisture by drying is a crucial aspect for accurately measuring mass loss due to dry sliding. A counterface disk fabricated from EN31 steel, with a hardness of 746 HV (62 HRC), was machined using 1000- and 2000-grit SiC abrasives. It was thereafter cleansed with acetone prior to and following each wear test and dried using a heated air stream. Wear testing was performed under dry sliding conditions utilizing a pin-on-disc (POD) tribometer, in accordance with ASTM G99, type (Ed-201 wear and friction monitor)/Ducom Make, Bangalore, India. All studies were conducted at 35 ± 1 °C. The assessments were performed in two cohorts. The initial set consists of four samples, which were examined for 15 min under applied loads of 10, 15, 20, and 25 N. The second group was subjected to a constant applied load of 25 N for 60 min. Each sample was measured using a precise digital balance (Sartorius AG, Göttingen, Germany) with an accuracy of 0.1 mg immediately before and after the wear test to produce (W_o_) and (W_1_), respectively. The fixed pin is oriented vertically on the rotating counterface surface. The wear rate was determined using the subsequent relationship [30,31]:

(4) $$WR=\rho_{pr}\times \frac{W}{S}$$

(5) S=V×t

(6) V=2πrn

(7) $$\Delta W=W_{o}-W_{1}$$

where n is sliding speed 470 rpm, r is radius of sliding 30 mm (from the center of the disk to the center of the sample), t is sliding period 15 min, S is sliding distance 1.329 × 10^6^ cm, V is velocity of linear sliding 1.4765 m·s^−1^, W_o_ is weight of unworn specimens (g), W_1_ is the weight of samples after the test (g), WR is the wear rate cm^3^/cm, and ρ_pr_ is the practically evaluated density of CoCrMo alloy and for the prepared nanocomposites (g/cm^3^).

All units have been combined in accordance with the applicable laws; consequently, to obtain the wear rate in cm^3^/cm units.

## 3. Results and Discussion

**Figure 1:**
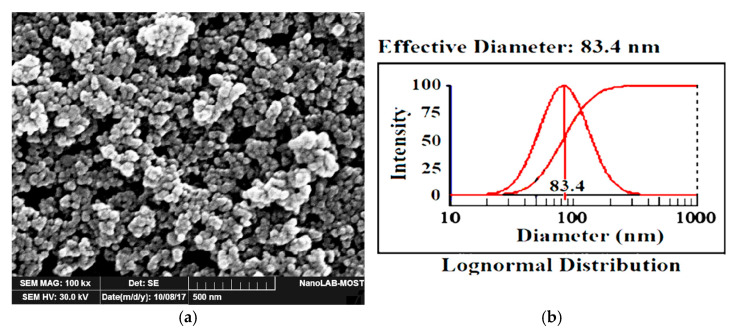
(**a**) SEM image for TiO_2_ nano-powder and (**b**) particle size distribution result diagram for TiO_2_ nano-powder.

**Figure 2:**
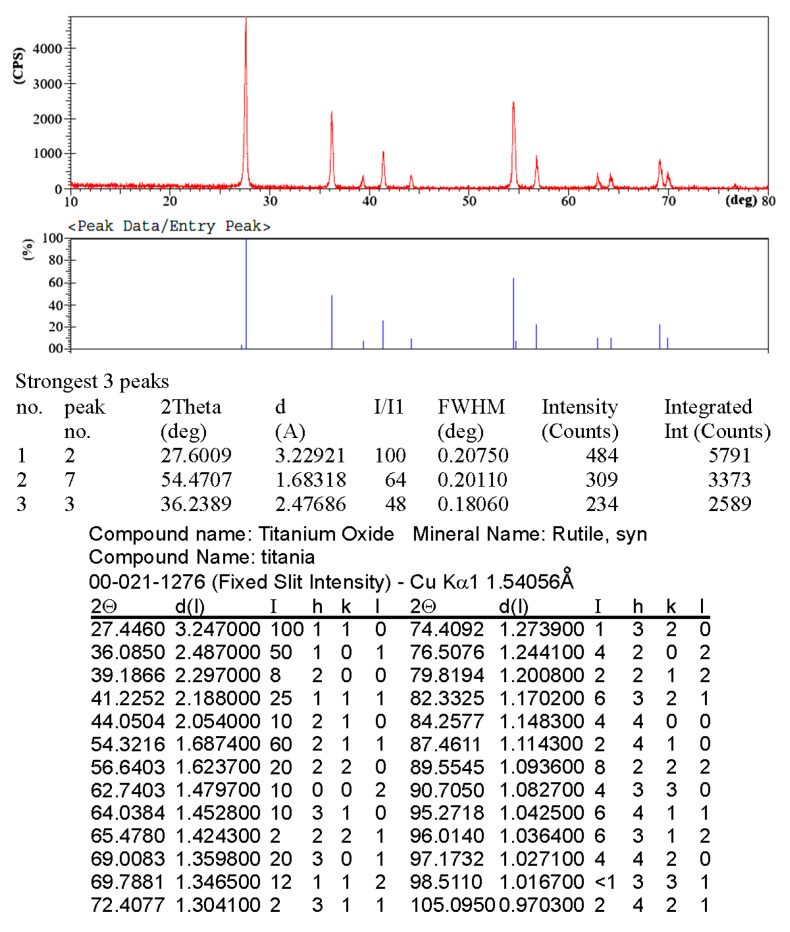
XRD analysis result for TiO_2_ nanoparticles.

Nano-TiO_2_ powder was observed with SEM as illustrated in Figure 1a. It manifests the semi-spherical shape of the particles and their presence somewhat as clusters. Additionally, the size of the nanoparticles is less than 100 nm. In turn, the particle size distribution (PSD) of the nano-TiO_2_ particles, as revealed in Figure 1b, indicates that the actual particle diameter is 83.4 nm. XRD test was performed for the XRD analysis graph for the use of nano-TiO_2_ particles, corresponding with rutile-type TiO_2_ card number 00-021-1276, as clarified in the table attached to the graph, as displayed in Figure 2.

**Figure 3:**
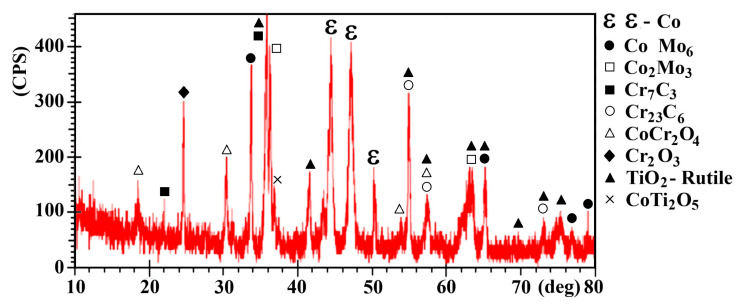
XRD analysis result for CCMA—5 wt% TiO_2_ nanocomposite.

Several phases are formed in the form of oxides such as Cr_2_O_3_, CoCr_2_O_4_, and CoTi_2_O_5_ that are illustrated in the XRD graph in Figure 3, which was due to the sintering process and also enhanced with the addition of nano-TiO_2_ to the base CCMA.

**Figure 4:**
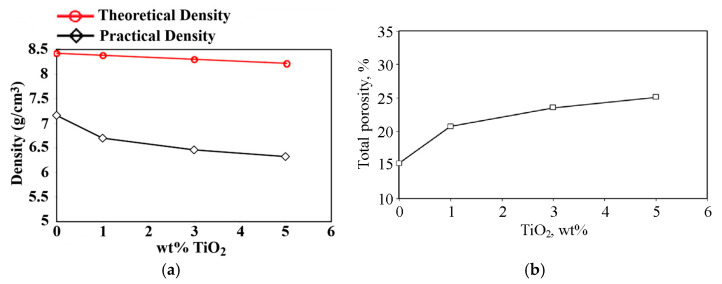
Variation of (**a**) theoretical and practical density and (**b**) total porosity of sintered CCMA at 1200 °C for 2 h due to TiO_2_ addition.

The theoretical and sintered CCMA density decreased when the TIO_2_ nanoparticle addition increased from zero to 5 wt%, as shown in Figure 4a. This is attributed to two causes. The first is that the products fabricated by PM are always associated with the creation of some amount of porosity, even after the application of high pressing force, and after completing the sintering process. The second is that the increase in the calculate porosity with the addition of nano-titania, as illustrated in Figure 4b, also affected by the reduction in the theoretical density of CCMA (with 8.415 gm/cm^3^) by addition of rutile type TiO_2_ NPs that has lower bulk density of TiO_2_ [32] (with 4.23 gm/cm^3^) as explained in Figure 4a.

**Figure 5:**
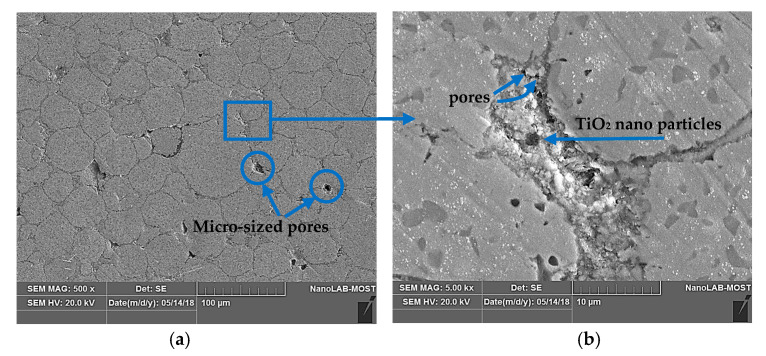
Microstructure of CCMA + 5 wt% TiO_2_ nanocomposites: (**a**) 500×, (**b**) 5000×.

The main resource of this porosity-raising value can be attributed, firstly, to the TiO_2_-NPs’ isolation role, which separates CCMA particles from each other, as shown in the SEM explanation in Figure 5a. This action has stopped the necking creation amongst most of the CCMA particles. Additionally, nano-titania particles, which occupy a reasonable fraction of the volume between the CCMA, in combination with their huge surface area, enhance the creation of a significant fraction of nano- and micro-sized pores among them, as illustrated in Figure 5a,b. Meanwhile, according to the fact that titania is a ceramic material, its particles were mechanically filled and pressed against each other, lacking diffusion among them, and with a small extent of chemically combining with CCMA particles forming the CoTi_2_O_5_ phase, despite being sintered at a 1200 °C temperature. Therefore, the number of pores will remain between them, and the factor that may reduce the porosity amount will depend mainly on the applied cold compacting pressure value. Due to this fact, a high cold pressing force was applied during the preparation of the samples in the present work. Nevertheless, an increase in the TiO_2_ addition to the base CCMA is illustrated in Figure 4b, which reduced the density of the CCMA by ~11.6% when the addition value became the maximum (i.e., 5 wt%), due to the lower bulk density of TiO_2_ with respect to the density of the CCMA powder. Nevertheless, a porous surface aids in mechanical interlocking among the nearby tissue and scaffolds. Moreover, the network structure of the pores aids in controlling and helping new tissue formation [33]. This former explanation indicates how porosity is an important beneficial factor when using the nanocomposite in biomedical applications.

**Figure 6:**
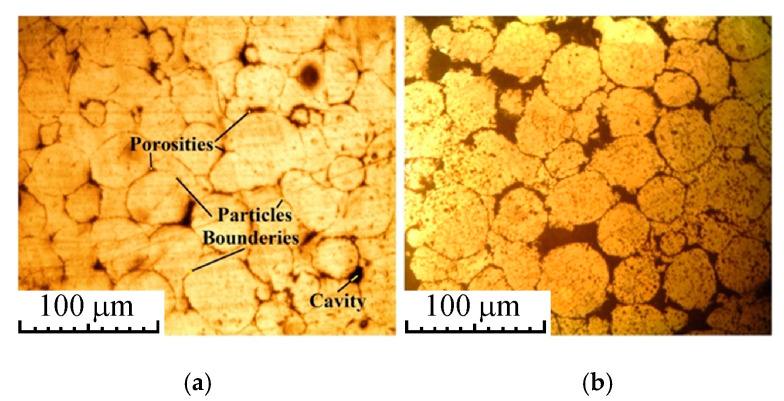

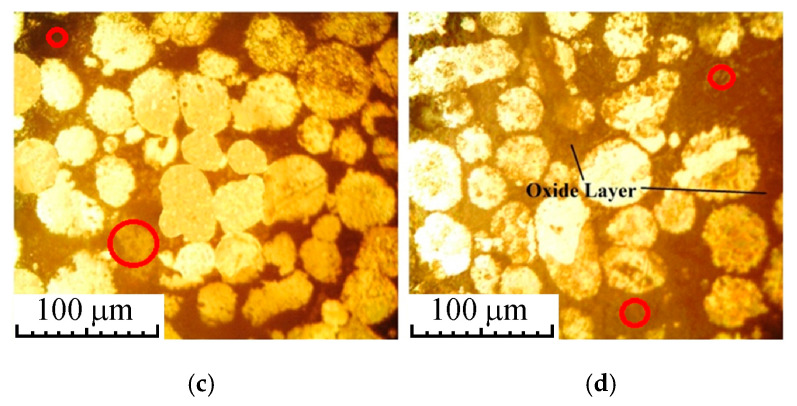
Microstructure of CCMA, which contains: (**a**) 0 wt%, (**b**) 1 wt%, (**c**) 3 wt%, and (**d**) 5 wt% nano-TiO_2_ particles.

From the point of view of optical observations, Figure 6a illustrates the remaining porosities, particularly at the junction between three adjacent particles, after completion of the pressing and sintering process. This is the actual case with products prepared by PM. Some of those pores are as large as so-called cavities that may be formed due to the absence of a small CCMA particle in its position, resulting from fragmentation during the surface preparation process by grinding and polishing for microscopic examination. Mainly, these defective positions appear as dark blackish zones. The addition of nano-TiO_2_ particles widened the interface zone between the CCMA particles in some positions and appeared as a brown-colored area, as illustrated in the b, c, and d sections of Figure 6. It is clearly observed that the brown-colored zones cover a greater area of the tested sample surface with an increase in the nano-addition fraction. Indeed, those positions are considered a mixture of chromium oxides, besides interacting nano-TiO_2_ particles at particle boundaries, with chromium oxides at other positions on some CCMA particles, as indicated by the red circles in Figure 6c,d.

**Figure 7:**
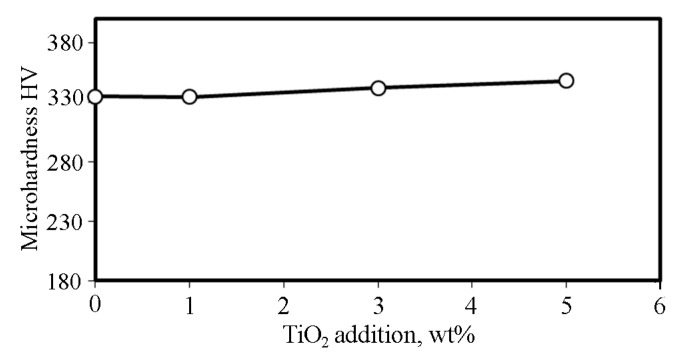
Effect of TiO_2_ addition on the microhardness of sintered CCMA.

Also, Levashov et al. [34] quantified that α-TiO_2_ nanoparticles increase from (0.92 to 3.3) wt%, which leaned towards increasing the porosity% from (5.8 to 16.1) for cobalt specimens prepared by hot-pressing. Although the addition of TiO_2_ contributed to increasing the measured total porosity, as shown in Figure 4b, the microhardness of sintered CCMA was slightly increased, as shown in Figure 7. This retention and a slight increase in the microhardness value (despite the increasing porosity with increasing NPs addition) belong to the formed Cr_2_O_3_, CoCr_2_O_4_, and CoTi_2_O_5_, which is illustrated in the XRD graph in Figure 3. As well as the added hard ceramic TiO_2_ NPs are responsible for compensating the inverse effect of increasing porosity with increasing NPs addition on the micohardness value. Where a part of the NPs embedded within CCMA particles then increased the hardness of the nanocomposite samples by a dispersion strengthening mechanism. The result was attaining the hardness of the CCMA, and with a slight increase at 5% TiO_2_ addition, is evidence of the success of the cold pressing process, where high compact pressure was applied, as well as the positive effect of the sintering process, which returns to the appropriate sintering temperature and time, along with adequate sintering inside an argon gas atmosphere.

**Figure 8:**
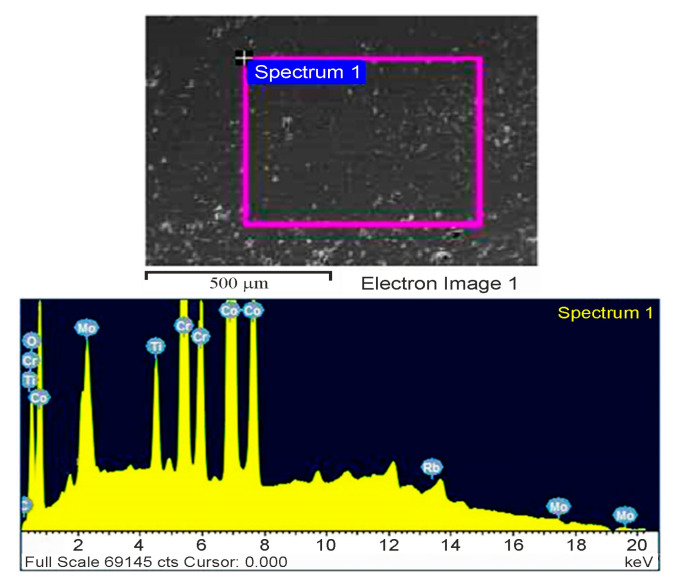
SEM-EDS analysis for CCMA + 3 wt% TiO_2_ nanocomposites.

Figure 8 illustrates the SEM-EDS analysis for the CCMA + 3 wt% TiO_2_ nanocomposites, where the presence of the titanium element was detected in the mapped area. It is crucial to clarify the method by which the added TiO_2_ nanoparticles were dispersed within the microstructure of CCMA particles, not only at their boundaries. This actionfacilitates understanding of the trends in the examined properties. Building on this guideline, the elemental dispersion of the fabricated composites was examined using the SEM-mapping facility.

**Figure 9:**
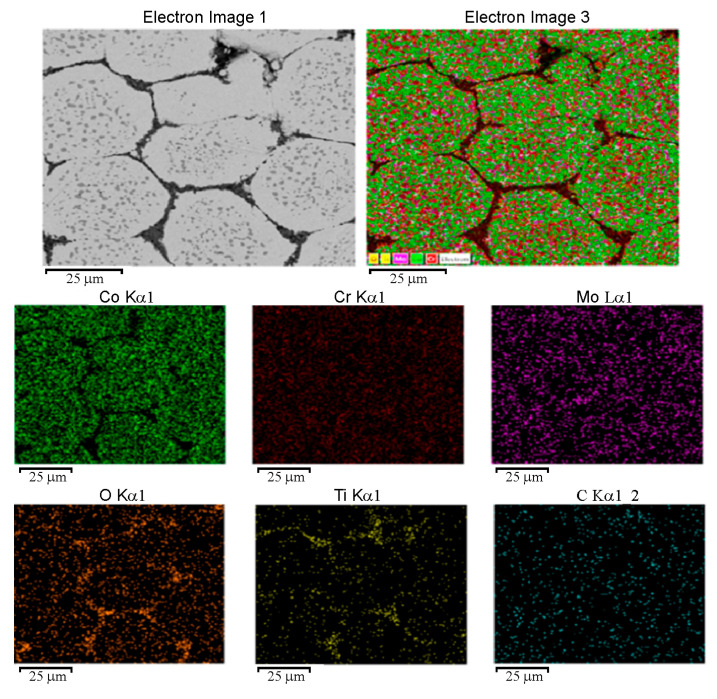

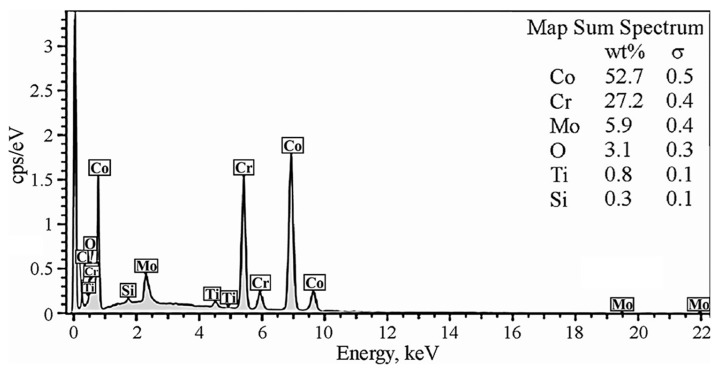
SEM-mapping outline and map sum spectrum for sintered CCMA + 3 wt% TiO_2_ nanoparticles.

The SEM-mapping profile of the CCMA-3 wt% TiO_2_ nanocomposite is shown in Figure 9. The appearance of Ti and O elements is greatly stabilized at CCMA particle boundaries, greater than within the CCMA particles. This is accurate evidence that TiO_2_ NPs are situated primarily at the CCMA particle boundaries, with a lower amount within the CCMA particles, as indicated by the yellow and brown color dots for Ti and O elements, respectively. It is important to indicate that the isolation of the CCMA particles by TiO_2_ NPs was performed mainly by mechanical separation, and to a lesser extent, due to chemical interaction between TiO_2_ and CCMA particles by the formation of CoTi_2_O_5_ oxide.

**Figure 10:**
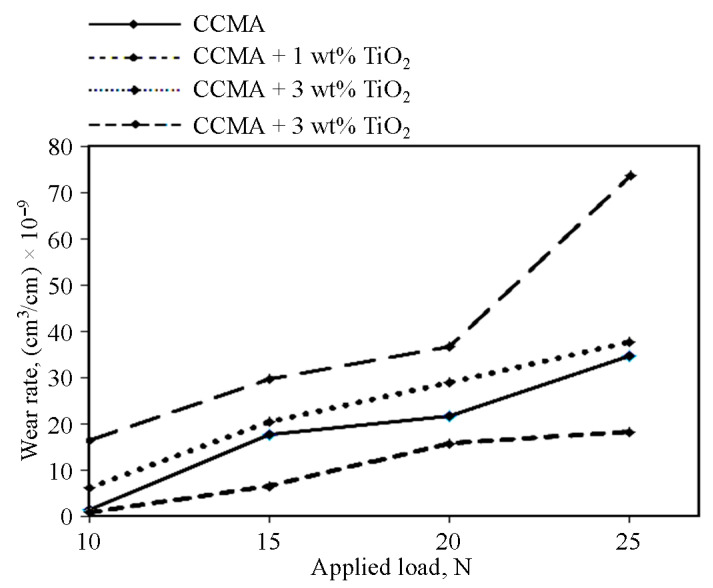
Dry sliding wear rates for CCMA-TiO_2_ nanocomposites.

Figure 10 shows the wear rate of the CCMA-TiO_2_ nanocomposites in the collection way, in comparison with CCMA. The nanocomposite containing 1 wt% TiO_2_ exhibited a lower wear rate than the base CCMA at all corresponding loads, indicating that although the total porosity of this nanocomposite increased slightly, its microhardness value remained approximately the same, thereby improving the wear resistance of CCMA according to the contiribbution of embeeded TiO_2_ NPs toward the resistance of the generated axial shear force on the tested sample surface due to dry sliding. An additional increase in the nano-TiO_2_ to 3 wt% and beyond resulted in a reduction in the wear resistance of CCMA, as evidenced by an increase in wear rate, shown in Figure 10.

**Figure 11:**
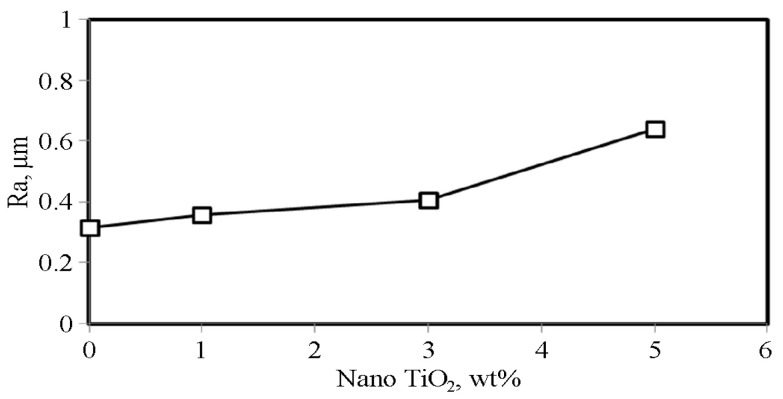
Measured surface roughness (Ra) for CCMA-TiO_2_ nanocomposites.

Besides the effect of the increased total porosity of the CCMA base alloy by raising the nano-TiO_2_ addition as maintained formerly, it is also reflected in the rise in the surface roughness of the sintered compacted samples, as illustrated in Figure 11. As a fundamental fact, the increase in roughness resulted in an increase in the number of asperities and an enlargement of their size. Accordingly, the amount of mass removed from the surface of the tested samples, in the form of debris particles, also increased. The addition of TiO_2_ NPs promotes tribo-oxidation due to an increase in Ra value. Hence, rising tribo-oxidation via uplifting local contact stresses and flash temperatures at asperity contacts, encouraging frequent oxide film falling-out and regeneration, then increasing exposure of fresh reactive surfaces, thereby quickening oxidation kinetics throughout sliding.

**Figure 12:**
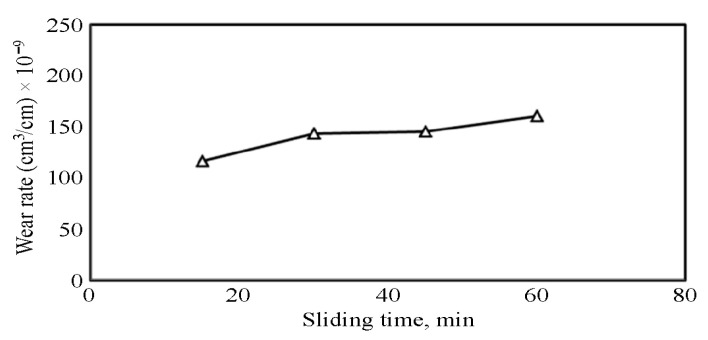
Dry sliding wear rate for CCMA + 5 wt% TiO_2_ nanocomposites.

**Figure 13:**
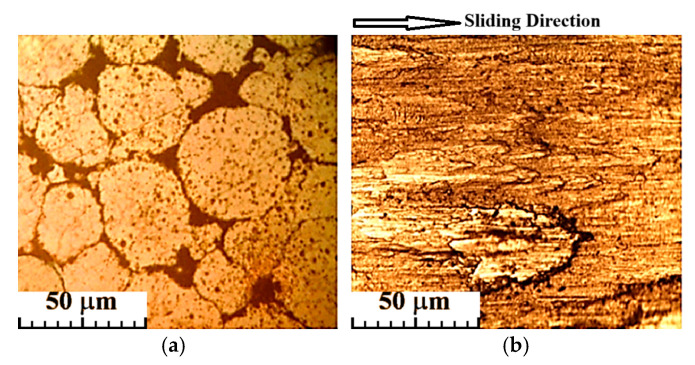
Change in the microstructure of the CCMA + 5 wt% of TiO_2_ nanocomposite: (**a**) before dry sliding and (**b**) after dry sliding.

**Figure 14:**
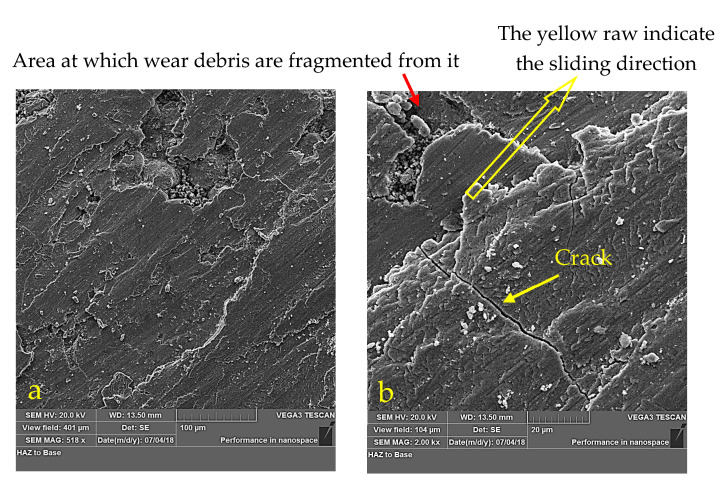
SEM image for worn surface appearance after dry sliding for 60 min under 25 N load for CCMA + 5 wt% TiO_2_ nanocomposites at different magnifications: (**a**) 518× and (**b**) 2000×.

The above explanation also applies to the variation in wear rate with time for the CCMA + 5 wt% TiO_2_ nanocomposites, as shown in Figure 12, which clearly indicates that the wear rate increased over time under dry sliding conditions. Figure 13 illustrates how the microstructure of the CCMA + 5 wt% TiO_2_ nanocomposite was changed after dry sliding. Where the particles’ real shape has disappeared, deteriorated, and elongated in the direction of sliding. The presence of rippled lines indicates exposure of the surface to plastic deformation in some sites, as shown in Figure 14a,b, which exemplify the worn surface of the CCMA + 5 wt% TiO_2_ nanocomposites under the same conditions listed above. The smooth wear scar tracks on the surface indicate the resistivity of CCMA alloy to the dry sliding at the operating conditions above. While the craze cracking on the surface, as clearly defined in Figure 14b, led to localized fractures in the surface and subsurface of the tested sample, and in turn, delamination and fragments of wear debris from the surface became easier.

Positions on the worn surface of the tested CCMA + 5 wt% TiO_2_ nanocomposites are also analyzed using area SEM-EDS and mapping to detect what happened from the perspective of surface chemistry.

**Figure 15:**
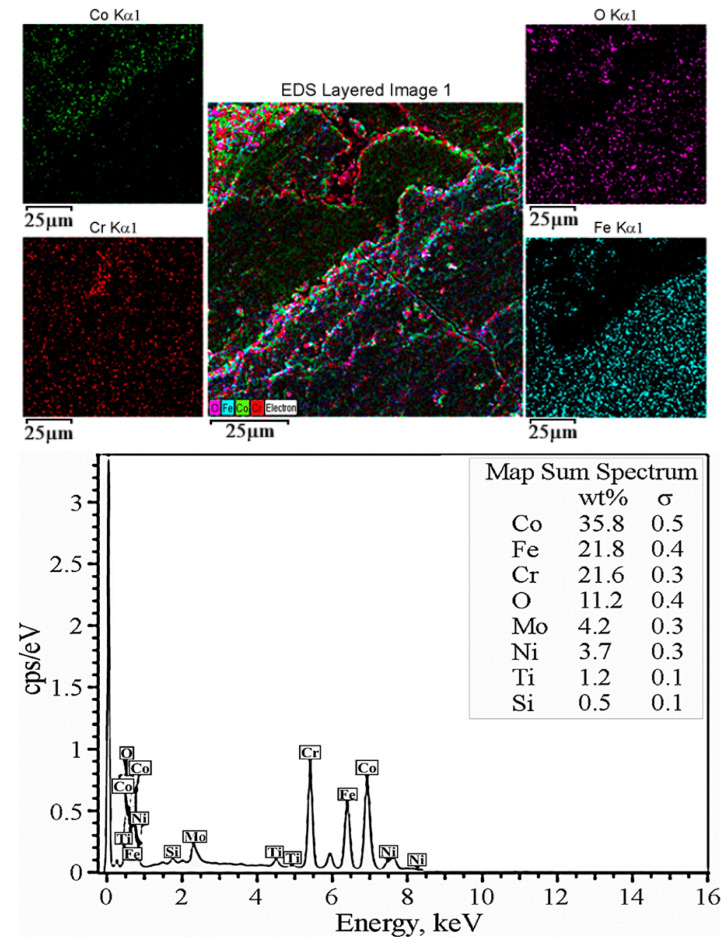
SEM-EDS -mapping analysis for worn surface appearance after dry sliding for 60 min under 25 N load for CCMA + 5 wt% TiO_2_ nanocomposites.

Figure 15 well describes the phenomenon of material transfer occurrence. The Fe element is distributed on the right-hand side and at other positions in the image. Also, the image reflects Co element depletion at the left-hand side of the mapped area. This means that the contact of the sample surface with the counterface was not uniform due to the dynamic changes in the roughness and topography of the sample surface. The applied load, combined with continuous dry sliding action, generated frictional heat, which in turn enhanced the transfer of Fe elements to the sample surface. The Ni element also exists in the map spectrum scan, which is one of the constituents of the counterface steel, ensuring the material transfer phenomena. The elemental layered topography variation, as illustrated in Figure 15, also serves as evidence of the continuous change in the microstructure of the worn surface and the generation of wear particle debris.

**Figure 16:**
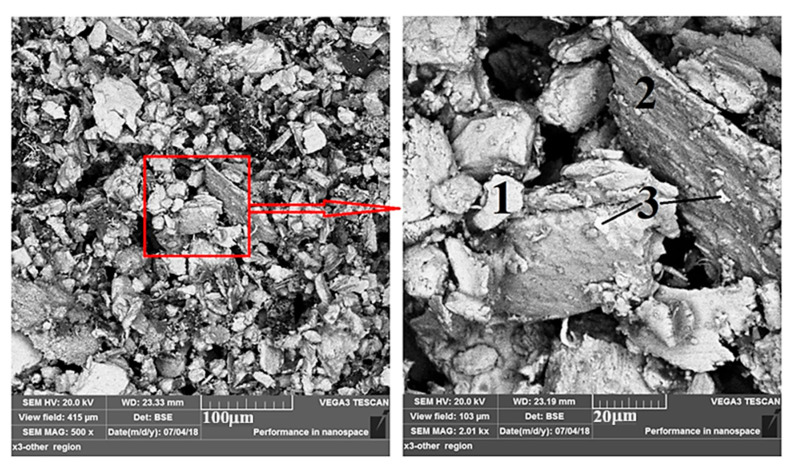
SEM images for the formed wear particle debris after dry sliding for 60 min under a 25 N load for CCMA + 3 wt% TiO_2_ nanocomposites.

Figure 16 illustrates an SEM image of the formed wear particle debris after dry sliding of the CCMA + 3 wt% TiO_2_ nanocomposite sample on the alloy steel counterface disk. Initially, the figure provides much important information about the formed debris. The formed debris has a wide range of sizes, spanning from less than 20 microns to slightly more than 60 microns. The debris is generated from the formed shear force created between the sliding couple, which in turn establishes the formation of cracks on the surface of the nanocomposite samples and propagates in different directions into the subsurface. Those cracks are connected after an interval of sliding, then promote the detachment of debris from their positions, leaving their sites as shallow or deeper depressions and cavities, as shown in Figure 14b. The magnified part of Figure 16 at the left-hand photo bound with red box is well illustrated at the left-hand photo in the same Figure 16 shows that the formed particles have irregular shapes, and their upper surface is smooth, as indicated by number 1, for example. On the other hand, the profile of the debris appeared as a rough texture, which was obtained because of the way the subsurface cracks propagated, as illustrated by number 2 in the magnified part of the same figure. The added nano-TiO_2_ particles, which indeed acted as a layer of coating positioned between CCMA particles, are strongly pinned on the CCMA particle surfaces where pieces of this coating remained connected to the surface of the debris particles even after wear debris particle detached from the tested sample, which appeared as a white colored position denoted by number 3 in the magnified section of Figure 16.

**Figure 17:**
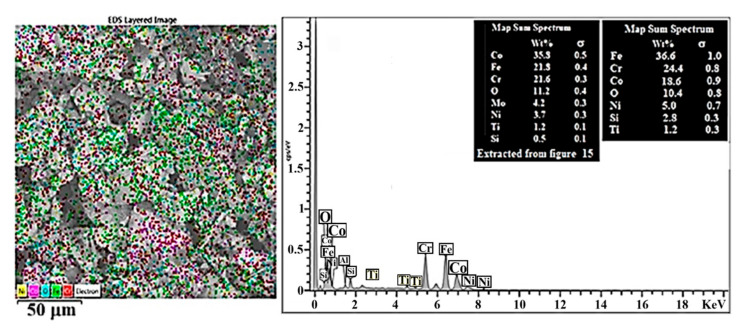
SEM-EDS area analysis for the formed wear particle debris after dry sliding for 60 min under a 25 N load for CCMA + 3 wt% TiO_2_ nanocomposites.

The SEM-EDS analysis of wear particles debris exemplifies a strong matching or agreement between the elements and their wt% that can be seen in the result listed in the black backgrounded table in Figure 17 with that extracted from Figure 14 that belongs to the SEM-EDS analysis result for the worm surface for the same sample (for easy comparison the elemental analysis table extracted from Figure 15 is beside the elemental analysis for the wear particle debris).

**Figure 18:**
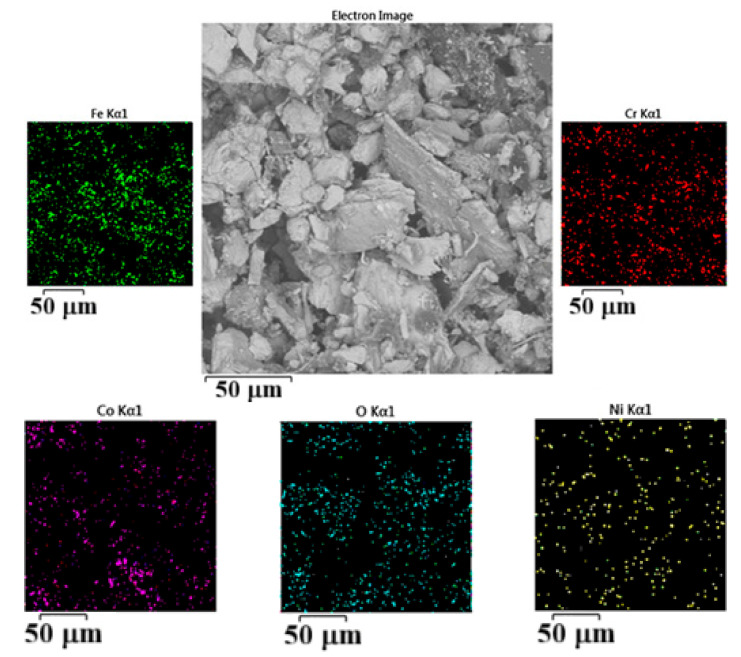
SEM-area mapping analysis for the formed wear particle debris after dry sliding for 60 min under 25 N load for CCMA + 3 wt% TiO_2_ nanocomposites.

Figure 18 depicts the five main elements that have the highest wt% presented within the mapped area of many wear particle debris. The result enhances the oxidation process that occurs during the dry sliding process on the sample surface being tested. Additionally, the material transfer phenomena are well explained by the presence of elements such as Fe and Ni, which are not detected in the chemical composition of the utilized CCMA powder. The two cases mentioned above exemplify the occurrence of dry sliding wear, primarily due to the adhesion mechanism.

The typical qualitative interpretation of the coefficient of variation (CV) shown in Table 1 gives a quick sense of how variable the data for studied properties are relative to the mean of their values. It is clear that the very low variability of the microhardness is due to the varying amount of added TiO_2_ NPs by means of the CV% value. This also indicates the very low standard deviation (σ) from the mean (μ) of the property. The moderate and high variability of porosity and surface roughness, respectively, for the nanocomposites prepared are closer to each other numerically, as indicated by the CV% values of them. This in turn describes how much the two properties are related to each other and can be controlled to fabricate more biocompatible nanocomposites, better than the biocompatibility known CCMA and TiO_2_ NPs, as far as these two properties are concerned.

## 4. Conclusions

Based on the research conducted, the following key conclusions can be drawn:

1. The nanocomposites are fruitfully made up utilizing CCMA with several additions of TiO_2_ nanoparticles via PMR.
2. No drop in the hardness of CCMA was detected when TiO_2_ nanoparticles were added. In contrast, its value increased slightly with the addition of nanoparticles.
3. The porosity was increased due to nanoparticle addition, and this will enhance the utilization of the nanocomposite in biomedical applications, such as bone fixation.
4. Even though TiO_2_ nanoparticles moderately isolated the pressed powder particles of CCMA, it does not stop the creation of Cr_2_O_3_ among the particles, which is revealed from the existence of Cr and O elements within the CCMA particles and at their boundaries.
5. The wear resistance of CCMA is improved at the lowest nano-TiO_2_ particle addition, while the wear rate increased and became more than that of the base CCMA; hence, if the wear resistance is the main requirement of the prepared nanocomposite, then the addition amount must be at the stated lowest quantity, which is 1 wt% TiO_2_ particle addition.
6. Wear particle debris analysis explained the formation of debris particles with different sizes and irregular shapes. Also, it indicated the creation of wear for the tested CCMA sample under the test conditions, mainly by the adhesion process.

## Figures and Tables

**Table 1:** Coefficient of variation (CV = σ/μ × 100) for some studied properties of the CCMA nanocomposites.

Property	μ	σ	CV%	Interpretation
Hardness	339.95	6.380178	1.877	Very low variability
Porosity	21.162	4.331567	20.461	moderate variability
Ra	0.448	0.154868	34.569	High variability

## Data Availability

The raw data supporting the conclusions of this article will be made available by the authors on request.

## Abbreviations

The following abbreviations are used in this manuscript:

CCMA	CoCrMo alloy
ρg	Green compact density (g/cm^3^)
EDS	Energy-dispersive X-ray spectroscopy
GC	Green compact
ρM	ρCo, ρCr, ρMo, ρSi, ρMn, and ρTiO_2_
Mc	Green compacted sample mass (gm)
NP	Nanoparticle
PMR	Powder metallurgy route
POD	Pin-on-disk
PSD	Particie size distribution
PTR	Powder technology route
SC	Sintered compact
SEM	Scanning electron microscopy
TP	Total porosity
Vg	Green compacted sample volume (cm^3^)
XM	(XCo, XCr, XMo, XSi and XMn) and TiO_2_%wt
XRD	X-ray diffraction
